# PUS1 is a novel biomarker for predicting poor outcomes and triple-negative status in breast cancer

**DOI:** 10.3389/fonc.2022.1030571

**Published:** 2022-11-15

**Authors:** Zheng Fang, Hong-yu Shen, Qi Xu, Hong-lei Zhou, Lei Li, Si-Yuan Yang, Zhen Zhu, Jin-hai Tang

**Affiliations:** ^1^ Department of General Surgery, the First Affiliated Hospital of Nanjing Medical University, Nanjing, China; ^2^ Gusu School, Nanjing Medical University, Nanjing, China

**Keywords:** PUS1, breast cancer, biomarker, prognosis, tissue microarray

## Abstract

Breast cancer patients’ outcomes have improved dramatically in recent years, but relapses and poor prognosis remain common due to its aggressiveness and heterogeneity. The development of reliable biomarkers is still needed for predicting prognosis and treatment effectiveness. Recently, a growing body of research suggests that pseudouridine synthases contribute to the development of many cancers, but their contribution to breast cancer remains largely unknown. Using an integrative analysis, we selected pseudouridine synthase1(PUS1) as the candidate biomarker. A tissue microarray of 131 breast cancer patients was then utilized to determine the clinical significance and prognostic value of PUS1. RNA sequencing analysis was conducted to identify downstream genes that differ between control and PUS1 knockdown cells. The effect of PUS1 on phenotypes of cells was assessed using cell proliferation, colony formation, and transwell invasion assays. We found that breast tumors overexpressed PUS1 compared with paired normal tissues. PUS1 expression was positively correlated with triple-negative breast cancer (TNBC) status (*P*= 0.020) and tumor grade (*P <*0.0001), but not with age (*P*= 0.736), tumor size (*P*= 0.608), lymph node (*P*= 0.742), oestrogen receptor (ER) (*P*= 0.162), progesterone receptor (PR) (*P*= 0.901), human epidermal growth factor receptor 2 (HER2) (*P*= 0.608) or tumor stage (*P*= 0.411). Comparatively, patients with high PUS1 levels had shorter overall survival time (*P*=0.0001) and relapse-free survival time (*P* = 0.0093). A univariate and multivariate survival analysis suggested that the overall survival of patients was independently influenced by the PUS1 score (Univariate Cox *P <*0.0001, HR=5.176, 95% CI =2.420-11.07; Multivariate Cox *P* = 0.001, HR = 5.291, 95% CI =1.893-14.78). RNA sequencing data revealed the PUS1 knockdown significantly affects a series of cancer related biological process such as regulation of cell proliferation and cell migration, as well as KEGG pathways including Mitophagy and PI3K-Akt signaling. *In vitro*, knockdown of PUS1 significantly suppressed the proliferation and colony formation abilities of MDA-MB-231 cells and BT-549 cells. Additionally, the ability of tumor cells to invade was remarkably attenuated in low PUS1 expression groups compared with the corresponding control groups. Our results suggested that PUS1 is a novel biomarker that predicts poor outcomes in patients with breast cancer and may prove to be a promising treatment target.

## Introduction

Breast cancer has been the most prevalent cancer with new cases diagnosed and approximately half a million women die from breast cancer each year (1). Even though outcomes among patient with breast cancer have improved dramatically recurrence, metastasis, and death are still frequently and are associated with poor prognosis. A part of the reason for this dismal prognosis could be its clinical characteristics of aggressiveness and high heterogeneity. Therefore, it is essential to comprehend the underlying pathophysiology and create trustworthy biomarkers in order to anticipate prognoses and treatment outcomes.

RNA modifications have been found the vital regulatory manners for the organisms to control gene expressions and provide vital links in various biological processes, including the development of human disease (2, 3). Pseudouridylation, a multi-step process involving post-transcriptionally mechanisms, is the second most abundant RNA modifications after m6A and exists in different RNAs including tRNAs, rRNAs, mRNAs. Pseudouridylation of RNA was produced either by the RNA independent mechanism or by a snoRNA dependent pathway (4). Recently, accumulating studies revealed that pseudouridine synthases implicate in the development of several cancers (5). For instance, the overexpression of Dyskerin pseudouridine synthase 1 (DKC1) was shown to be significantly correlated with unfavourable clinicopathological parameters and poor prognosis of breast cancer (6). High expression of pseudouridine synthase 7 (PUS7) prognosticates the poor outcome of individuals with glioblastoma and inhibition of PUS7-mediated pseudouridine modification restrained the glioblastoma stem cell (GSC) tumorigenesis (7). Additionally, PUS7 regulates its downstream effector LIM and SH3 protein 1(LASP1), which promotes colorectal cancer metastasis (8). However, clinical significance and cellular functions of pseudouridine synthases in breast cancer are not well understood.

As an important member of pseudouridine synthase family, pseudouridine synthase1(PUS1) can regulate the Pseudouridylation of almost all kinds of RNAs including mRNAs, snoRNAs, tRNAs, ncRNAs and snRNAs (9). It has been reported that PUS1 regulate the Pseudouridylation of mRNA by recognize the special structure of mRNA instead of specific sequences (4, 10). In this study, we screened the expressions of pseudouridine synthase family members in breast cancer by an integrative analysis and choose PUS1 as the candidate biomarkers for this tumor. Based on tissue microarray analysis, we further evaluated PUS1’s clinical significance as well as its correlations with patients’ prognosis. An RNA sequencing method was used to explore how PUS1 regulates breast cancer cells’ biological processes, which was then validated by *in vitro* experiments.

## Material and methods

### Bioinformatic analysis of the cancer genome atlas, gene expression omnibus datasets and clinical proteomic tumor analysis consortium datasets

the tcga rna-seq data for invasive breast cancer (brca) from the GDC (Genomic Data Commons) portal (http://portal.gdc.cancer.gov/) and five microarray datasets of breast cancer (GSE5364, GSE22820, GSE42568, GSE45827 and GSE65212) from GEO database (www.ncbi.nlm.nih.gov/geo/) were downloaded, analyzed using the “Bioinformatics analysis” module of the Home-for-Researchers website (https://www.home-for-researchers.com/static/index.html#/) and the Sangerbox bioinformatic tools (http://www.sangerbox.com/tool). The “CPTAC” module of the UALCAN database (http://ualcan.path.uab.edu/index.html) was queried for the comparison of proteomic data of candidate pseudouridylate synthases in breast cancer (11).

### Human tissue samples and cell lines

Declaration of Helsinki was followed in all areas of this study. First Affiliated Hospital of Nanjing Medical University (Ethics code 2021-SR-308) provided ethical clearance. Specimens of breast cancer were collected from Nanjing Medical University’s First Affiliated Hospital’s Department of General Surgery, and patients or their next of kin provided informed consent. The enrolled patients were diagnosed with breast cancer according to clinical symptoms, physical examination, imaging and histopathological diagnosis, and the excluded patients were those with unresectable, metastatic breast cancer, other malignant tumors such as breast sarcoma and malignant lymphoma of the breast, benign breast diseases, and those with severe cardiovascular and renal diseases who were unable to receive radical surgery. None of the patients received preoperative chemotherapy or radiotherapy. Liquid nitrogen was used to snap 12 paired freeze breast tumors and normal tissues following excision of the surgical specimen. **Supplementary Table 1** contains the catalog number of samples used for western blot assay. A tissue microarray with 131 breast cancer tissues was purchased from Shanghai Outdo Biotech Company (Shanghai, China). Cell lines used in this study included one normal mammary epithelial cell line (MCF10A) and ten breast cancer cell lines representing the genomic features of main breast cancer subtypes (Luminal subtype: MCF-7, MDA-MB-415, ZR-75-1; Her2 positive:SK-BR-3, HCC1954; TNBC : MDA-MB-453, MDA-MB-231, BT-549, Hs-578T, HCC1937. All the cell lines were derived from the Cell Bank of Shanghai Academy of Chinese Sciences. MCF-10A cells were maintained in DMEM/F-12 supplemented with 5% horse serum, hrEGF (20 ng/ml), hydrocortisone (0.5 μg/ml), cholera toxin (100 ng/ml), and insulin (10 μg/ml). RPMI-1640 supplemented with 10% fetal bovine serum was used for cell culture at 37°C in 5% CO2. Once the cells had reached 80–90% confluency, they were digested with Trypsin-EDTA, centrifuged for five minutes at 500g, resuspended, and counted using a haemocytometer (Thermo Fisher Scientific, MA, USA). Then the cells were cultured in a culture dish of 60 x 15 mm or seeded in 96 well plate for further research.

### Immunohistochemistry of tissue microarrays

The tissue microarrays were stained with the IHC kit (KIT-9710, Maixin, China). We baked the microarrays for an hour at 60°C, dewaxed them in xylene, and rehydrated them in decreasing amounts of ethanol in accordance with manufacturer’s instructions. After treated with endogenous enzymes blocking reagents and nonspecific blocking reagents, the slices were incubated with PUS1 antibody diluted 1:4000 (ab203010, Abcam) overnight. The next day, the pathological slice was sequentially incubated with donkey anti-mouse/rabbit secondary antibodies and Streptomyces anti-biotin protein-peroxidase for 10 minutes. Chromogenic detection was accomplished using the DAB Detection Kit (DAB-2031, Maixin, China) and the section was counterstained with hematoxylin. Finally, the tissue microarrays (TMA) were dehydrated using a gradient concentration of ethanol and xylene. and sealed with neutral resin. Two independent pathologists examined the immunohistochemistry staining and had no prior knowledge of the patient’s characteristics. The intensity of PUS1 staining was evaluated on three levels (negative = 0; weak = 1; medium = 2; strong = 3) and four categories were established for the percentage of tumor cells that were positive: 0, <5% positive tumor cells; 1, 5–25% positive tumor cells; 2, 26–50% positive tumor cells; 3, between 51–75% positive tumor cells; 4, > 75% positive tumor cells.

### Western blot assay

Protein extracts from tissues and cells were obtained by adding RIPA lysis buffer containing PMSF, protease inhibitors, and phosphatase inhibitors. Using a BCA Protein Assay Kit (Beyotime, China), the protein concentration was determined. We electrophoresed 20ug protein per lane using 10% SDS-PAGE and transferred it to PVDF membranes (Millipore, 0.45um) at 110V for 60 minutes. Afterward, the gel was transferred to PVDF membranes (Millipore, 0.45um) at 200mA for 60 minutes. In 15 minutes, the membranes were blocked with QuickBlockTM Western blocking solution (Beyotime, China) at room temperature and subsequently incubated overnight at 4 degrees Celsius with rabbit anti-PUS1 diluted 1:1000 (ab203010, Abcam) and mouse anti-GAPDH diluted 1:10000 (10494-1-AP, Proteintech). Incubation with secondary antibodies was carried out for 1 hour following three TBST washes at room temperature. The membranes were then washed three additional times with TBST and incubated with enhanced chemiluminescence (ECL) Plus (Yeasen Biotechnology, China), which was imaged using a Bio-Rad ChemDoc XRS (Bio-Rad, USA). The densities of the blots were measured using ImageJ.

### Short hairpin RNA vectors and stable knockdown of PUS1 in breast cancer cells

A puromycin-resistant pLKO.1 vector was used to design the shRNA vectors targeting PUS1, and shRNA sequences are listed in **Supplementary Table 2**. In order to produce viral preparations, Lipofectamine was used to transfect lentiviral vectors and packaging plasmids (PsPAx and PMD2G) into 293T cells. The viral supernatants were collected 48 to 72 hours after transfection and added to breast cancer cells treated with polybrene at 0.01 mg/ml. Following puromycin selection at a dose of 2 ug/mL for seven days, the cells were maintained with puromycin and then prepared for the next stage of identification by western blot assay.

### RNA sequencing assay and data analysis

PUS1 knockdown and control groups were compared using RNA sequencing to find downstream genes that were differentially expressed. We extracted total RNA from shPUS1 knockdown and control groups of MDA-MB-231 cells using Trizol reagent (Invitrogen, CA, USA) according to the manufacturer’s instructions. VAHTS mRNA‐seq V2 Library Prep Kit for Illumina (NR601, Vazyme) was used to construct the library. For the preparation of the following libraries, 1 ug of total RNA was used and isolation of poly(A) mRNA was performed using Oligo(dT) beads. Using divalent cations and high temperatures, mRNA fragmentation was achieved and the priming process was carried out using random primers. Two strands of cDNA were synthesized, one from the first strand and one from the second strand. As soon as the double-stranded cDNA was purified, adaptors were added to both ends after dA tailing and T-A ligation. DNA Clean Beads were then used to select the size of adaptor-ligated DNA. Amplification of each sample was then conducted using P5 and P7 primers, and the obtained products were validated by PCR. Following that, the Illumina Novaseq 6000 was used to analyze libraries with different indexes. Sequencing was done using 2x150 paired-end (PE) configuration in accordance with manufacturer’s instructions. The RNA sequencing assays were performed in triplicates. For data analysis, a reference genome sequence of human species and gene model annotation files were downloaded and indexed, and a transcript profiling analysis was performed using Hisat2 software for alignment to reference genomes. General feature format (GFF) annotation files are converted to fasta format for expression analysis. HTSeq (v0.6.1) calculated the gene and isoform expression levels based on the pair-end clean data and the reference gene file. To analyze differential expression, the DESeq2 Bioconductor package was used and a list of enriched genes with *p* value less than 0.05 was identified using “ClusterProfiler”.

### Cell vialibity and colony formation assay

Using a 96-well plate, 3,000 cells were plated per well in triplicate and viability was measured after 0, 24h, 48h, 72h, 96h. The results were examined by adding 100ul of fresh RPM1640 containing 10ul of CCK-8 assay solution to each well. A plate reader was used to measure 450 nm absorbance. An assay for colony formation was conducted by plating 2000 cells in a 6 cm dish and culturing them for 10 days. Following a PBS wash of the dishes, fixations were conducted in 4% paraformaldehyde for 15 minutes, followed by 30 minutes of Chrystal Violet staining. The colonies were examined under a microscope to determine if they had at least 50 cells.

### Transwell invasion assay

We conducted an invasion assay by adding 1:10 diluted matrix gel to serum-free RPM1640 medium and incubating transwell inserts for 24 hours at 37°C. Following that, the insert with matrix gel on the bottom was placed in a well of a 24 well plate, A suspension of 8x10^5^ cells in 200ul serum-free medium was plated into the inserts (6.5mm Diameter, 8.0 μm pore size, NEST 725301, China), which were then placed in a well containing 700ul complete medium. After 24 hours, cells in the upper chambers were removed. For staining, 4% paraformaldehyde was applied to the undersurface cells for 20 minutes, followed by 15 minutes of 0.1% crystal violet. Under a microscope, five randomly selected areas were counted

### Statistical analysis

An analysis and mapping of the data was performed using GraphPad Prism 8.0 (USA, San Diego). Comparing the means of two groups was done using *t*-tests, and a chi-square test was used to determine the relationship between PUS1 expression and clinical parameters. By using Kaplan-Meier analysis, we compared the overall survival rate and the relapse free survival rate and a univariate and multivariate survival analysis was run using R packages “survival” and “survminer”. Significant results were determined by a *P* value of 0.05.

## Results

### Breast cancer tissues showed high PUS1 mRNA and protein expression as compared to normal tissues.

**Figure 1:**
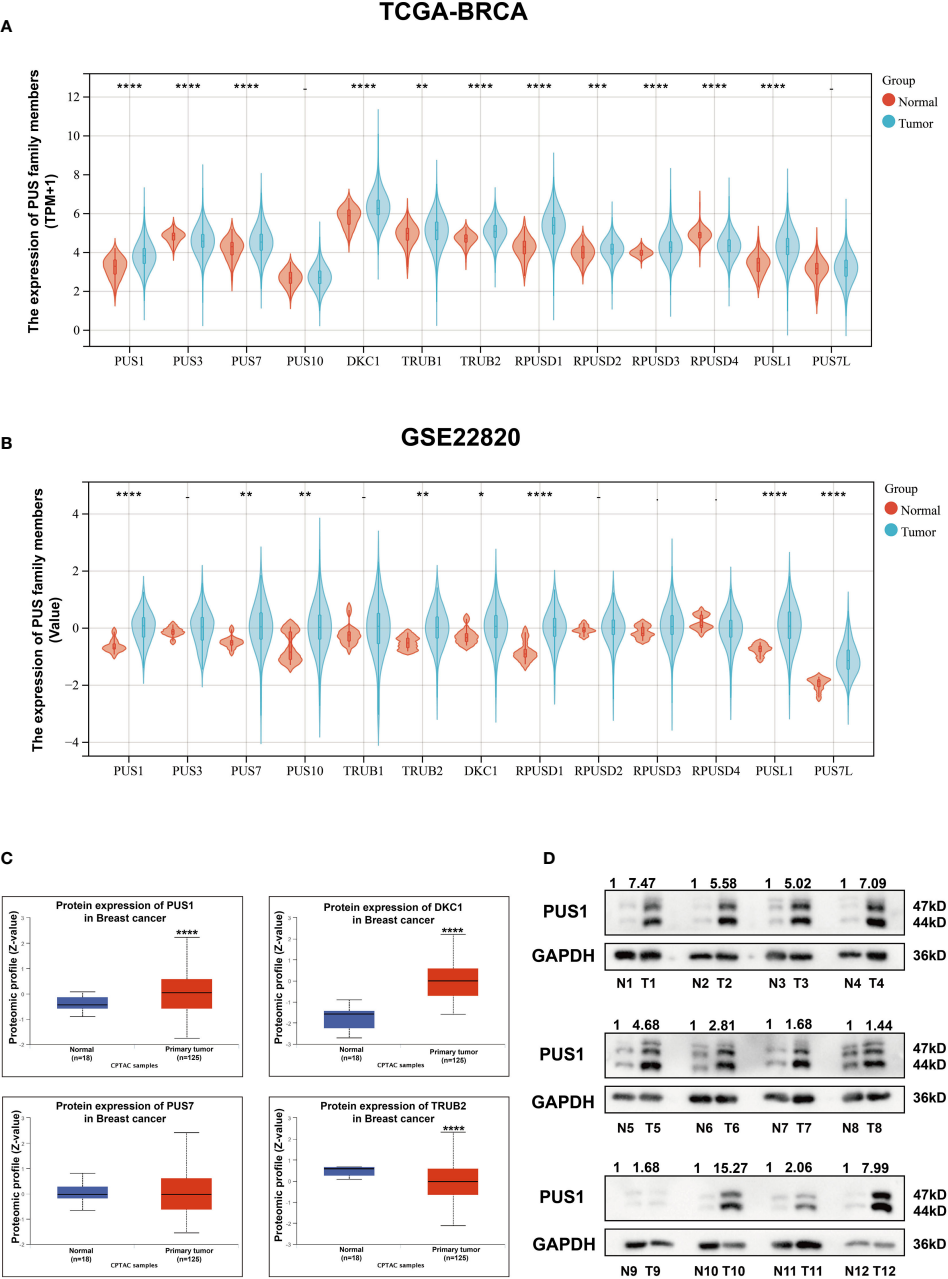
There was higher expression of PUS1 mRNA and protein in breast cancer tissues than in normal tissues. **(A, B)**; The mRNA expression values for individual members of the pseudouridylate synthase family in TCGA and GEO22820 datasets are plotted in violins. Red violins represent normal tissues and green violins tumor tissues.; **(C)** The proteomic results of the four pseudouridylate synthases are displayed as z-values based on the CPTAC breast cancer dataset generated by UALCAN database. Blue, normal tissue. Red, primary tumors. The median Z values of PUS1 and DKC1 in primary tumors were substantially greater than those in normal tissues while the median of TRUB2 was lower in tumor tissues than in healthy tissues. No significant difference of PUS7 was observed between the tumor and normal groups. **(D)** The protein expression of PUS1 in 12 paired breast cancer tissues and normal tissues validated by western blotting assay. The relative intensity of bands was first measured and qualified by ImageJ, and then normalized to GAPDH. Detection of PUS1 revealed two bands with approximately molecular masses of 44 and 47 kDa and most tumor tissues showed higher levels of PUS1 than their corresponding normal tissues. *, *P* value < 0.05; **, *P* value < 0.01; *** or more, *P* value < 0.001.

To validate the family members of pseudouridylate synthases in BRCA, we first analyzed their mRNA levels in five GEO breast cancer datasets and TCGA-BRCA cohort based on 1097 breast cancer samples and 113 normal breast tissues. As shown in **Figures 1A, B** and **Supplementary Figure 1**, all selected datasets indicated higher levels of PUS1, PUS7, TruB pseudouridine synthase family member 2 (TRUB2), and DKC1 in tumors compared with normal tissues. Then we analyzed their protein levels in breast cancer by querying the proteomic expression profiles from CPTAC, as shown in **Figure 1C**, the medians of PUS1and DKC1 were significantly higher in primary tumors than those in normal tissues(PUS1, *P <*0.0001; DKC1, *P <*0.0001) while PUS7 and TRUB2 showed the contradictive trends with their mRNA expressions in BRCA. It is worth noting that DKC1 is also a telomerase which is difficult to be a therapeutic target due to the undesirable cytotoxic effects on stem cells (12, 13). Considering the wide range of RNA targets modified by PUS1 and the direct modification pattern carried out by this member, we choose PUS1 for further investigation (4). Further western blot assays were carried out on paired breast tumors and adjacent normal tissues, and **Figure 1D** shows that most breast tumors exhibited higher expression of PUS1 than normal tissues in accordance with proteomics results.

### PUS1 positively correlated with TNBC and tumor grade status, and had a predictive value for poor prognosis

**Figure 2:**
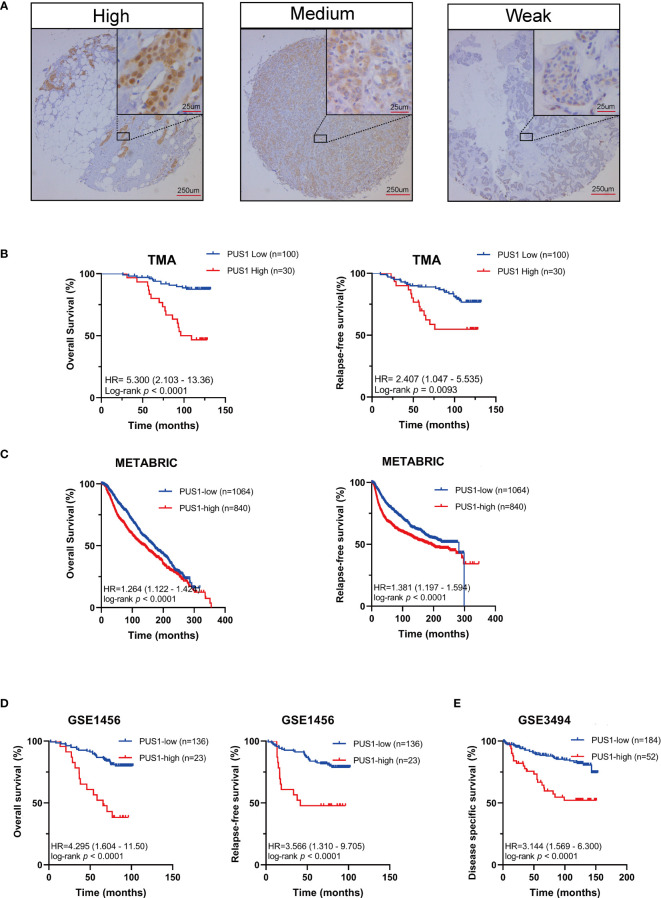
UP-regulation of PUS1 correlated with poor prognosis for patients with breast cancer **(A)** Representative immunohistochemical staining of PUS1 in breast cancer TMA. Brown staining in the nucleus or plasma indicates positive staining. **(B)** Kaplan–Meier curves analysis of the protein level of PUS1 and the survival of breast cancer patients from the TMA cohort. Red, PUS1 high expression group. Blue, PUS1 low expression group. High PUS1 protein levels were linked to the patients’ poor overall survival and relapse-free survival. **(C, D)** The correlation between mRNA expression level of PUS1 and overall survival as well as relapse-free survival in the METABRIC and GSE1456 datasets. At the mRNA level, high PUS1 expression in both cohorts predicted patients’ adverse outcomes. **(E)** Kaplan–Meier curves showing the disease-specific survival stratified by PUS1 mRNA expressions in the GSE3494 dataset. TMA, tissue microarray.

In order to determine the clinical significance of aberrant PUS1 expression in breast cancer, we evaluated PUS1 expression by immunohistochemistry in tissue microarrays from 131 patients. According to **Figure 2A**, tumor cells expressed PUS1 primarily in the nucleus and plasm. We calculated the IHC score for each specimen and divided the patients into high PUS1(N=35) and low PUS1 subgroup(N=96). As shown in **Table 1**, a positive correlation was found between the PUS1 expression TNBC status (*P*= 0.020) and tumor grade (*P <*0.0001), but not with age (*P*= 0.736), tumor size (*P*= 0.608), lymph node (*P*= 0.742), ER (*P*= 0.162), PR (*P*= 0.901), HER (*P*= 0.608) or tumor stage (*P*= 0.411). As compared to those with low PUS1 levels, patients with high PUS1 levels had shorter overall survival (HR=5.300, *P* =0.0001) and relapse-free survival (HR= 2.407, *P* =0.0093) (**Figure 2B**). For a deeper exploration of PUS1’s predictive role in breast cancer, we conducted univariate and multivariate analyses on our cohort, and found the overall survival of patients was independently influenced by the PUS1 score (Univariate Cox *P <*0.0001, HR=5.176, 95% CI =2.420-11.07; Multivariate Cox *P* = 0.001, HR = 5.291, 95% CI =1.893-14.78) (**Table 2**). By analyzing three public datasets, we further validate our conclusions concerning the aberrant PUS1 mRNA level and patient survival. Accordingly, high PUS1 mRNA levels predict poor overall survival and relapse-free survival in METERBRIC (HR=1.264, P <0.0001; HR=1.381, P <0.0001) and GSE1456 datasets (HR=4.295, *P <*0.0001; HR=3.566, *P <*0.0001) as well as adverse disease specific survival in GSE3494 dataset (HR=3.144, *P <*0.0001), showing that PUS1 is an unfavorable biomarker for breast cancer (**Figures 2C–E**).

**Table 1:** Clinicopathological associations of PUS1 IHC score.

Variables		PUS1 Score		x^2^	*P* value
	N	high	low		
**Patient age**					
**≤50**	48	12 (34.29%)	36 (37.50%)	0.1141, 1	0.736
**>50**	83	23 (65.71%)	60 (62.50%)		
**Tumour size**					
**≤2 cm**	61	15 (42.86%)	46 (47.92%)	0.2639, 1	0.608
**>2 cm**	70	20 (57.14%)	50 (52.80%)		
**lymph nodal status**					
**negative**	68	19 (54.29%)	49 (51.04%)	0.1081, 1	0.742
**positive**	63	16 (45.71%)	47 (48.96%)		
**ER status**					
**positive**	88	21 (60%)	67 (72.83%)	1.960, 1	0.162
**negative**	39	14 (40%)	25 (27.17%)		
**Missing**	4				
**PR status**					
**positive**	56	15 (42.86%)	41 (44.09%)	0.01560, 1	0.901
**negative**	72	20 (57.14%)	52 (55.91%)		
**Missing**	3				
**Her2 status**					
**positive**	23	5 (15.15%)	18 (19.15%)	0.2632, 1	0.608
**negative**	104	28 (84.85%)	76 (80.85%)		
**Missing**	4				
**TNBC status**					
**non TNBC**	96	20 (60.61%)	76 (80.85%)	5.426, 1	**0.020**
**TNBC**	31	13 (39.39%)	18 (19.15%)		
**Missing**	4				
**tumor stage**					
**I−II**	86	21 (60%)	65 (67.71%)	0.6758, 1	0.411
**III**	45	14 (40%)	31 (32.29%)		
**tumor grade**					
**II**	110	21 (60%)	89 (92.71%)	20.39, 1	**<0.0001**
**III**	21	14 (40%)	7 (7.29%)		

The P value in bold indicates that the P value is statistically significant (P value < 0.05).

IHC, immunohistochemistry; ER, oestrogen-receptor; PR, progestin receptors; Her2, human epidermal growth factor receptor 2; TNBC, triple negative breast cancer.

**Table 2:** Univariate and multivariate cox regression analysis of prognostic factors in breast cancer patients.

Variables	OS Univariate		OS Multivariate		RFS Univariate		RFS Multivariate	
	HR (95%CI)	*P* value	HR (95%CI)	*P* value	HR (95%CI)	p value	HR (95%CI)	*p* value
**PUS1 score**	5.176 (2.420-11.070)	**<0.0001**	5.291(1.893-14.780)	**0.001**	2.299 (1.165-4.537)	**0.016**	1.097 (0.436-2.756)	0.843
**Age**	2.435 (0.925-6.407)	0.071	4.777 (1.633-13.96)	**0.004**	1.282 (0.616-2.671)	0.506	1.728 (0.791-3.772)	0.17
**Tumor size (≤2cm vs>2cm)**	1.354 (0.634-2.892)	0.433	1.359 (0.575-3.209)	0.483	1.184 (0.606-2.314)	0.62	0.672 (0.310-1.459)	0.316
**Grade (IIvsIII)**	2.820 (1.275-6.238)	**0.01**	1.535 (0.520-4.530)	0.438	2.527 (1.211-5.272)	**0.013**	3.237 (1.290-8.119)	**0.012**
**N (N0 vsN1-3)**	2.434 (1.101-5.382)	**0.028**	3.791 (0.840-17.10)	0.083	2.726 (1.334-5.570)	**0.006**	0.479 (0.058-3.922)	0.493
**Stage (T1-2 vs T3)**	2.809 (1.328-5.942)	**0.007**	1.001 (0.259-3.866)	0.998	4.592 (2.280-9.248)	**<0.0001**	9.746 (1.273-74.57)	**0.028**
**Her2 status (negative vs positive)**	1.236 (0.498-3.062)	0.647	1.410 (0.406-4.897)	0.589	1.352 (0.612-2.987)	0.455	1.431 (0.495-4.135)	0.508
**ER status (negative vs positive)**	0.268 (0.125-0.572)	**0.001**	0.266 (0.071-0.995)	**0.049**	0.363 (0.187-0.705)	**0.003**	0.300 (0.088-1.021)	0.054
**PR status (negative vs positive)**	0.695 (0.328-1.469)	0.341	0.856 (0.354-2.073)	0.732	1.124 (0.578-2.187)	0.729	1.864 (0.823-4.218)	0.135
**TNBC (nonTNB vs TNBC)**	3.170 (1.489-6.747)	**0.003**	1.126 (0.251-5.050)	0.876	2.273 (1.137-4.543)	**0.02**	1.038 (0.270-3.984)	0.956

The P value in bold indicates that the P value is statistically significant (P value < 0.05). IHC, immunohistochemistry; ER, oestrogen-receptor; PR, progestin receptors; Her2, human epidermal growth factor receptor 2; TNBC, triple negative breast cancer; OS, overall survival; RFS, Relapse-free survival. HR, hazard ratio. CI, confidence interval.

### PUS1 might involve in tumor progression by influencing a series of cancer related biological processes and pathways

**Figure 3:**
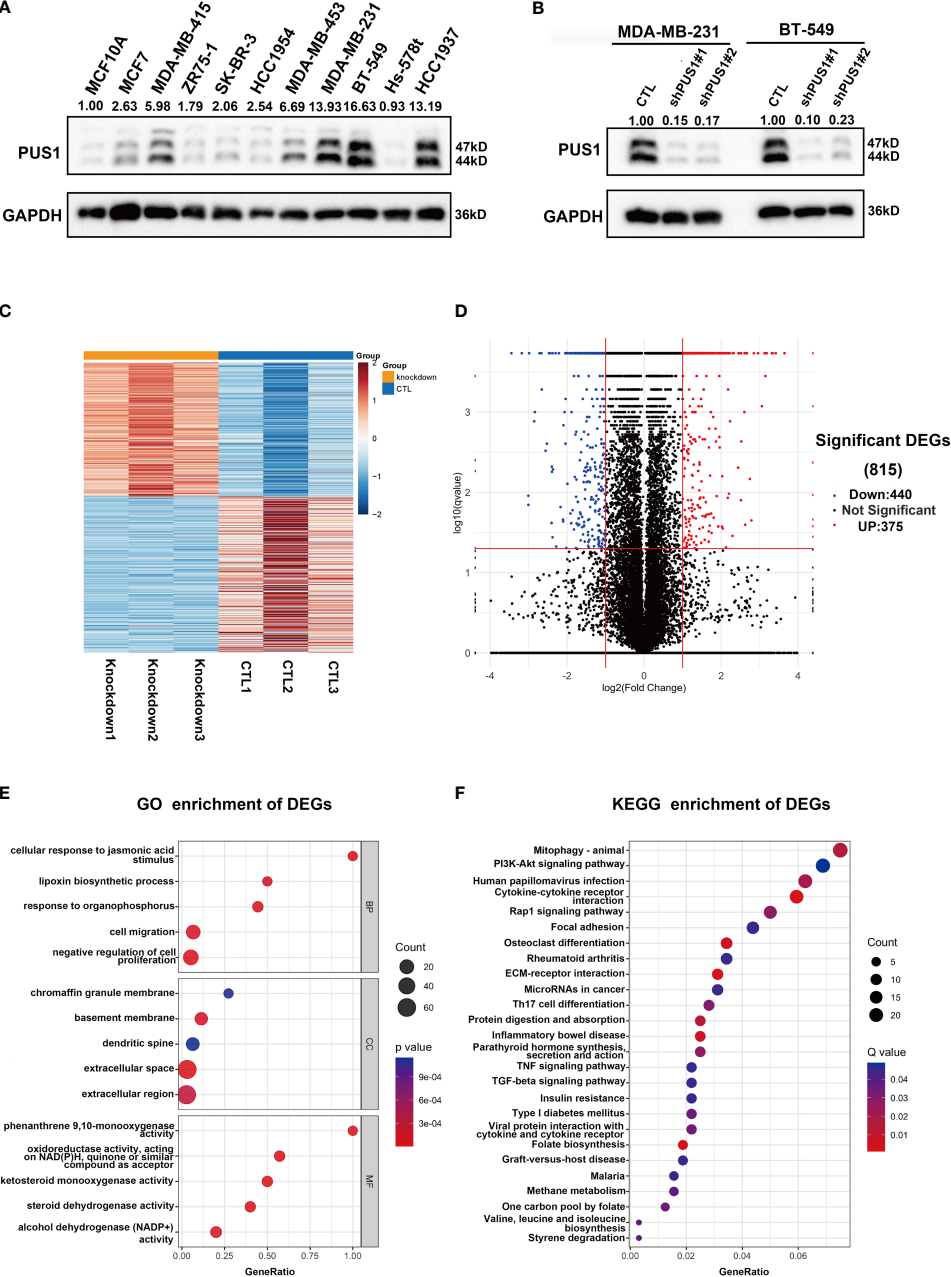
PUS1 might involve in tumor progression by influencing a series of cancer related biological process and pathways. **(A)** Relative protein level of PUS1 in normal mammary epithelial cell MCF10A and a series of breast cancer cell line validated by western blot assay. The breast cell lines representing the genomic features of the main breast cancer subtypes were selected, including luminal MCF-7, MDA-MB-415, ZR-75-1; Her2 positive, SK-BR-3, HCC1954 and triple negative subtype MDA-MB-453, MDA-MB-231, BT-549, Hs-578T, HCC1937. Three TNBC cell lines relative strongly expressed PUS1 including MDA-MB-231, BT-549 and HCC1937. **(B)** The stable silencing of PUS1 expression by lentivirus infection in MDA-MB-231 and BT-549 cells. Western blot analysis showed that the protein level of PUS1was remarkably knocked down in the shPUS1 groups compared with CTL groups. CTL, cells transfected with control shRNA lentivirus; shPUS1, cells transfected with PUS1 shRNA lentivirus. **(C, D)** The heatmap and volcano plot of DEGs between the PUS1 knockdown MDA-MB-231 cells and negative control group **(E, F)** The GO enrichment and KEGG enrichment Analysis of the DEGs between PUS1 knockdown high and low expression groups. DEGs, differentially expressed genes. GO, Gene ontology. KEGG, Kyoto Encyclopedia of Genes and Genomes.

Our first step in analyzing PUS1’s phenotypic impact on breast cancer is to assess the relative protein level of PUS1 in breast cancer cells. According to **Figure 3A**, MDA-MB-231 BT-549 and HCC1937 cells expressed relatively higher levels of PUS1 than other breast cancer cells as well as normal mammary epithelial cell line. Then by transfecting MDA-MB-231 and BT-549 cells with lentiviral vectors, we achieved stable knockdown of PUS1, and PUS1 protein expression was remarkably decreased in shRNA-PUS1 group compared with shRNA-control group (**Figure 3B**). Subsequently, we performed RNA-seq analysis of PUS1 knockdown MDA-MB-231 cells and control groups. The PUS1 knockdown group had 444 genes significantly down-regulated and 375 genes significantly up-regulated compared to the control group (**Figures 3C, D**). A Gene Ontology (GO) analysis was conducted to investigate the functions of downstream genes of PUS1 and the biological processes related to a series of cancer related signature such as cell proliferation and cell migration (**Figure 3E**). We also performed KEGG pathway analysis and found the differentially expressed genes (DEGs) enriched in several cancer pathways such as Mitophagy, PI3K-Akt signaling pathway, Cytokine-cytokine receptor interaction, Rap1 signaling pathway, and focal adhesion, suggesting that a wide range of crucial cancer cell characteristics can be influenced by PUS1 through a complex regulatory network during tumor progression (**Figure 3F**).

### MDA-MB-231 and BT-549 cells exhibited decreased cell viability and colony formation when PUS1 was knocked down

**Figure 4:**
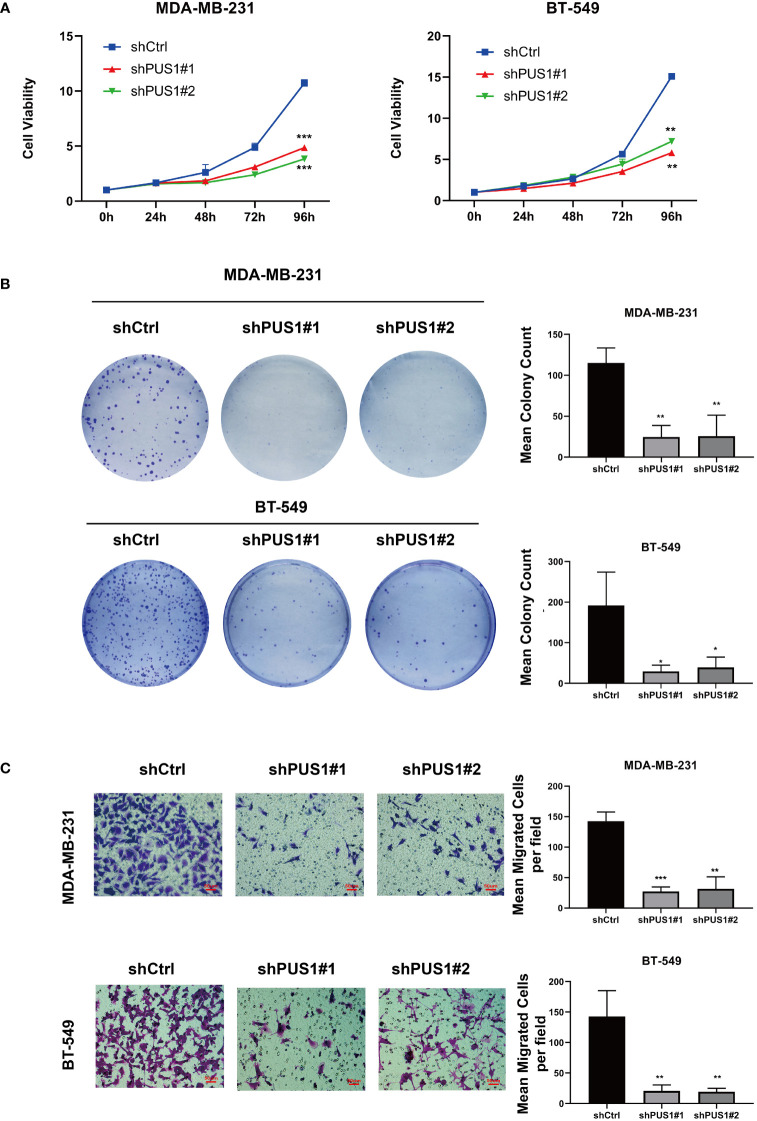
MDA-MB-231 and BT-549 cells that were knocked down for PUS1 showed decreased cell viability, colony formation, and invasion of tumor cells. **(A)** The cell viability of MDA-MB-231 and BT-549 determined by CCK-8 assay. The relative viability of tumor cells was significantly suppressed in the shPUS1 groups relative to the negative control groups. **(B)** The colony formation abilities of two cell lines examined by colony formation assay. The colonies formed by tumor cells was considerably fewer in the shPUS1 groups than in the control groups. **(C)** Transwell invasion assay showed that the number of tumor cells which migrated from the upper to lower chamber was significantly reduced in the shPUS1 groups compared with the control groups. The data were expressed as the means ± standard deviation with three repeats. *, P value < 0.05; **, *P* value < 0.01; ***, *P* value < 0.001.

The first step in examining the impact of PUS1 on the phenotype of cells was to compare the viability of cell cultures between shPUS1 and control groups. As determined by CCK-8 tests, MDA-MB-231 and BT-549 cells showed dramatic reductions of cell viability after PUS1 knockdown (**Figure 4A**). Then we tested whether PUS1 knockdown affected breast cancer cells’ long-term survival capacity, and the number of colonies formed by tumor cells was also significantly diminished at low PUS1 levels (**Figure 4B**). The results of our study confirmed that MDA-MB-231 and BT-549 cells are less proliferative and colony forming when PUS1 is downregulated.

### MDA-MB-231 and BT-549 cells invaded less when PUS1 was knocked down

By conducting a transwell invasion assay, we further examined the impacts of PUS1 on cell invasion. It can be seen in **Figure 4C** that significantly fewer MDA-MB-231 and BT-549 cells migrated from the upper to lower chamber in PUS1 knockdown groups than in control groups. This evidence suggested that knocking down PUS1 decreased breast cancer cells’ ability to invade, which was consistent with our bioinformatic analysis of the RNA sequencing data.

## Discussion

According to our findings, breast cancer tissues showed a greater expression of PUS1 than normal tissues. A high expression of PUS1 correlated with TNBC status and higher tumor grade, as well as poor prognosis of patients with this cancer. *In vitro*, downregulation of PUS1 affects a variety of cancer related pathways. As a result of knocking down PUS1 *in vitro*, MDA-MB-231 and BT-549 cells grew slower and invaded less.

Pseudouridylation of RNA is widespread in the prokaryotes and eukaryotes. However, its functions have been underappreciated for a long time until the recent discovery that it greatly contributes to efficacy of the mRNA COVID-19 Vaccines (14, 15). Derived from uridine by base-specific isomerization, pseudouridylation modification was generally considered to stabilize the RNA structure, increase the protection of the RNA against nucleases and affects the translation of mRNA into protein (9). In view of the widespread effects RNA pseudouridylation has on RNA metabolism and gene expression, it is not surprising that pseudouridylation influenced the progress of oncogenesis (16). Intriguingly, the pseudouridine synthases, which “write” pseudouridylation modifications, play a dual role in malignant diseases, acting as both tumor promoters and tumor suppressors (17). In current study, we examined the aberrant expression of PUS1 in breast cancer as well as its clinical significance and prognostic value. It is worth noting here that we validated the prognostic value of PUS1 by using a breast tissue microarray which is capable of achieving a rapid IHC staining of large sample size with small batch effects. We also found that positive correlation between high PUS1 expression and unfavorable pathological parameters including higher tumor grade and TNBC status, which represent the higher degrees of malignancy and more aggressive biological behavior of breast cancer. In line with the PUS1 level in breast cancer tissues, PUS1 were highly expressed in most TNBC cell lines compared with the normal mammary epithelial cell and other tumor cell lines. According to our knowledge, these findings have not been reported in the literature till now. Notably, we must emphasize that despite the possibility that high PUS1 RNA levels might also predict poor outcomes which supported by several public datasets, IHC detection of PUS1 is more stable, reliable, and realistic for clinical applications in the future.

**Figure 5:**
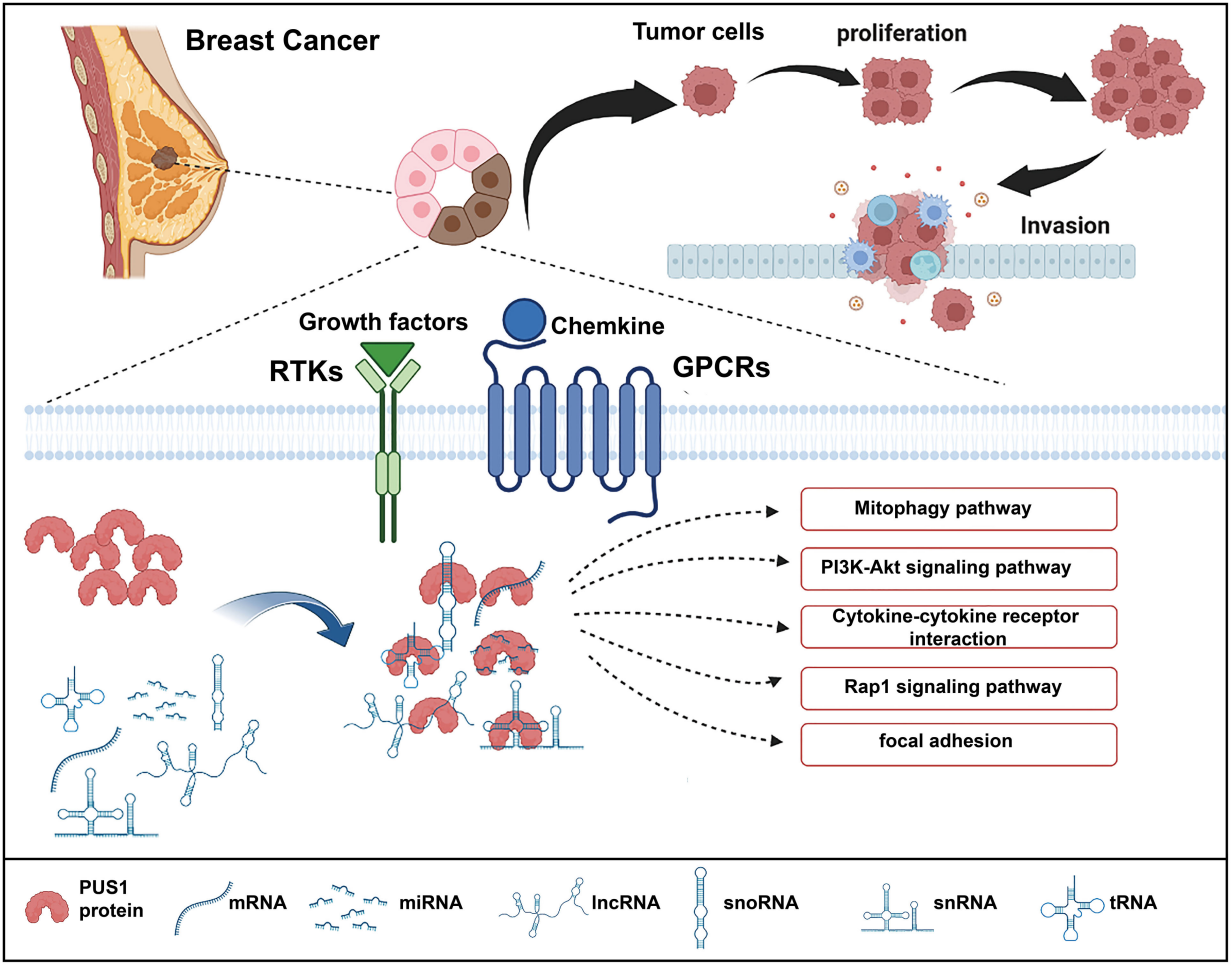
Schematic illustration of the functions of PUS1 and its potential regulation mechanisms in breast cancer. Upregulation of PUS1 promotes malignant biological behavior of tumor cells by regulating a series of cancer related biological processes and pathways.

As high expression of PUS1 correlates with poor prognosis, we hypothesize that PUS1 is a tumor-promoting gene in breast cancer. To this date, there is no knowledge of the regulation network mediated by PUS1 that contributes to cancer progression. Therefore, we performed RNA sequencing to analyze the downstream genes impacted by PUS1 knockdown and speculated the possible impacts induced by PUS1. We found that PUS1 regulated many cancer-related biological process including cell proliferation and cell migration. We also noticed that PUS1 affect many crucial pathways including Mitophagy, PI3K-Akt signaling pathways which were frequently deregulated in cancers. Mitophagy appears to have a controversial role for there is some evidence that mitophagy defects promote breast cancer bone metastasis, while others say a mitophagy inhibitor makes breast cancer cells more susceptible to chemotherapy (18, 19). The PI3K-Akt pathway is strongly correlated with cell proliferation, survival, invasion, and migration in many cancers, and it is one of the most frequently activated pathways in breast cancer (20–22). Based on our enrichment analysis, we propose a possible mechanism for PUS1 in promoting breast cancer progression (**Figure 5**).

It has not been reported in previous study that PUS1 impacted the cell phenotype of cancer cells. We found PUS1 knockdown suppressed tumor proliferation and invasion in accordance with our RNA sequencing results. On the other hand, we also found some support for our results in previous studies. For example, GSC lines showed drastic growth suppression after knockdown of PUS7 (7). Additionally, downregulation of DKC1 inhibited prostate cancer cell growth and glioma cell invasion (23, 24). The results of our study provide further evidence that pseudouridine synthases are crucial in the progression of cancer as well. Therefore, it is possible that PUS1 could serve as a future therapeutic target for breast cancer.

There were some pitfalls and drawbacks in present study. First, each spot of the microarray originated from just one part of the tissue and it might be difficult to represent the full view occasionally considering the high degree of tumoral heterogeneity. To further validate how aberrant PUS1 expression influences breast cancer risk, more clinical samples would be required from different cohorts. Second, the phenotypes of PUS1 knockdown on tumor cells was observed *in vitro* which need to validated on *in vivo* experiments in future. Third, this study sought to investigate whether PUS1 has clinical significance in breast cancer as well as how it affects phenotypic features of cells, but the direct RNA substrates of PUS1 as well as the complicated roles of pseudouridylation modifications on breast cancer has not been explained. Further studies should be applied to find the direct targes of PUS1 by using a series of methods such as RIP-sequencing and pseudouridine sequencing. More importantly, small molecule inhibitors which specifically acts on PUS1 and its downstream targets should be screened to accelerate translational clinical research.

In conclusion, our results suggested the great potential of PUS1 as an unfavorable biomarker for breast cancer. Cancer cells are significantly inhibited in their ability to proliferate, form colonies, and invade when PUS1 is inhibited. PUS1 might be a promising treatment target for breast cancer.

## Data availability statement

The datasets presented in this study can be found in online repositories. The names of the repository/repositories and accession number(s) can be found below: https://www.ncbi.nlm.nih.gov/geo/, GSE212074.

## Ethics statement

The studies involving human participants were reviewed and approved by the First Affiliated Hospital of Nanjing Medical University Medical Science Research Ethics Committee(Ethics code 2021-SR-308). The patients/participants provided their written informed consent to participate in this study.

## Author contributions

Conception and design of the work: ZZ and J-HT. Acquisition, analysis, and interpretation of data: ZF, HS, QX, HZ, LL and S-YY. Drafting and revising of the article: ZF, ZZ and J-HT. Final approval of the manuscript and agreement to be accountable for all aspects of the work: All authors. All authors contributed to the article and approved the submitted version.

## Funding

This research was supported by the National Key Research and Development Program of China (No. 2016YFC0905900), National Natural Science Foundation of China (No. 81872365) and Jiangsu Provincial Key Research Development Program (No. BE2019731).

## Acknowledgments

The authors would like to gratefully acknowledge all the patients and their families who contributed specimens to the study, and the surgical staff who assisted in the collection of specimens.

## Conflict of interest

The authors declare that the research was conducted in the absence of any commercial or financial relationships that could be construed as a potential conflict of interest.

## Publisher’s note

All claims expressed in this article are solely those of the authors and do not necessarily represent those of their affiliated organizations, or those of the publisher, the editors and the reviewers. Any product that may be evaluated in this article, or claim that may be made by its manufacturer, is not guaranteed or endorsed by the publisher.
