# Supplementary figures and images for: PUS1 is a novel biomarker for predicting poor outcomes and triple-negative status in breast cancer
Source: Front Oncol. 2022 Nov 15;12:1030571. doi: 10.3389/fonc.2022.1030571 (PMC9705787; doi:10.3389/fonc.2022.1030571)

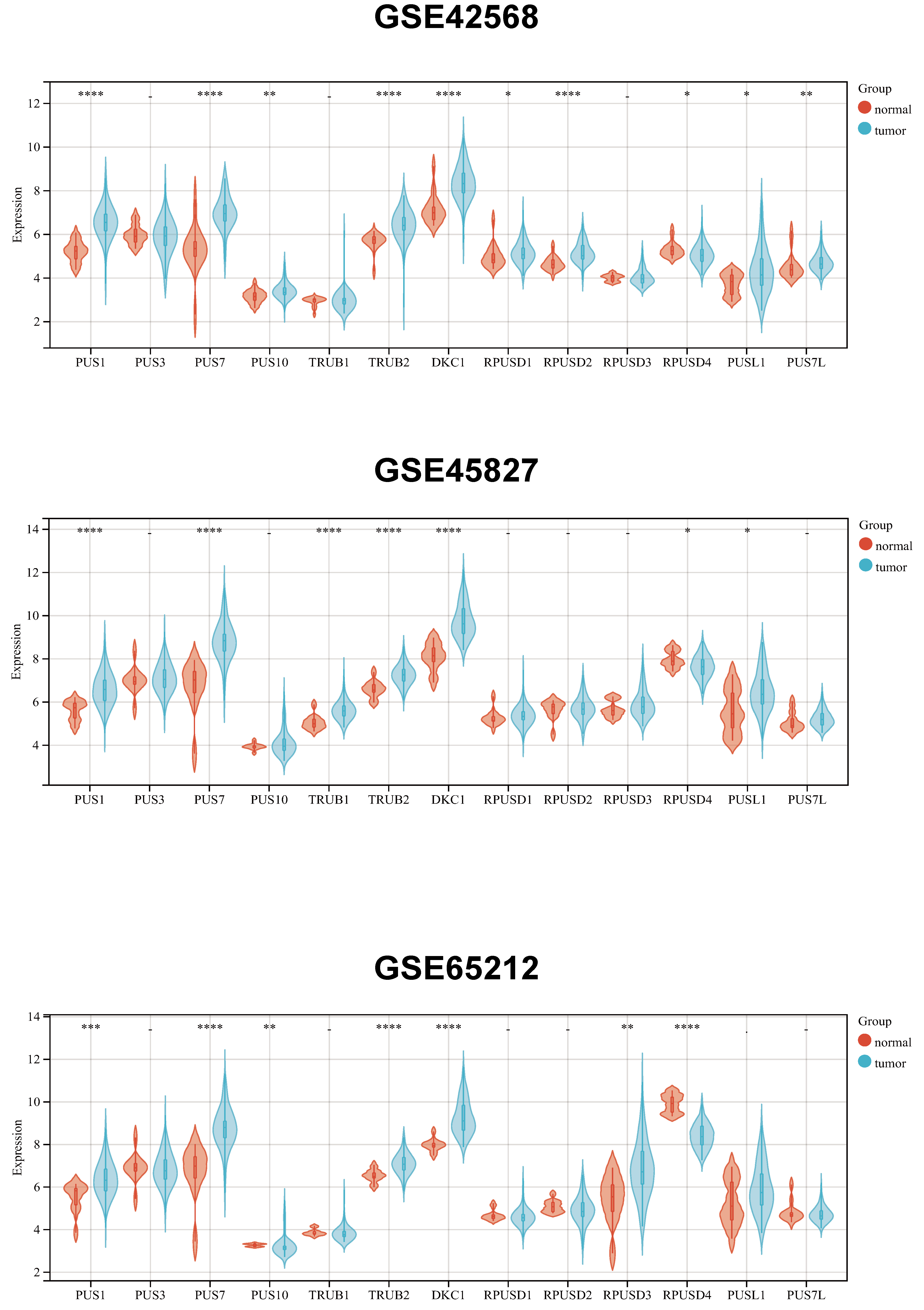

Supplement: Supplementary file 1 [file Image_1.jpeg]

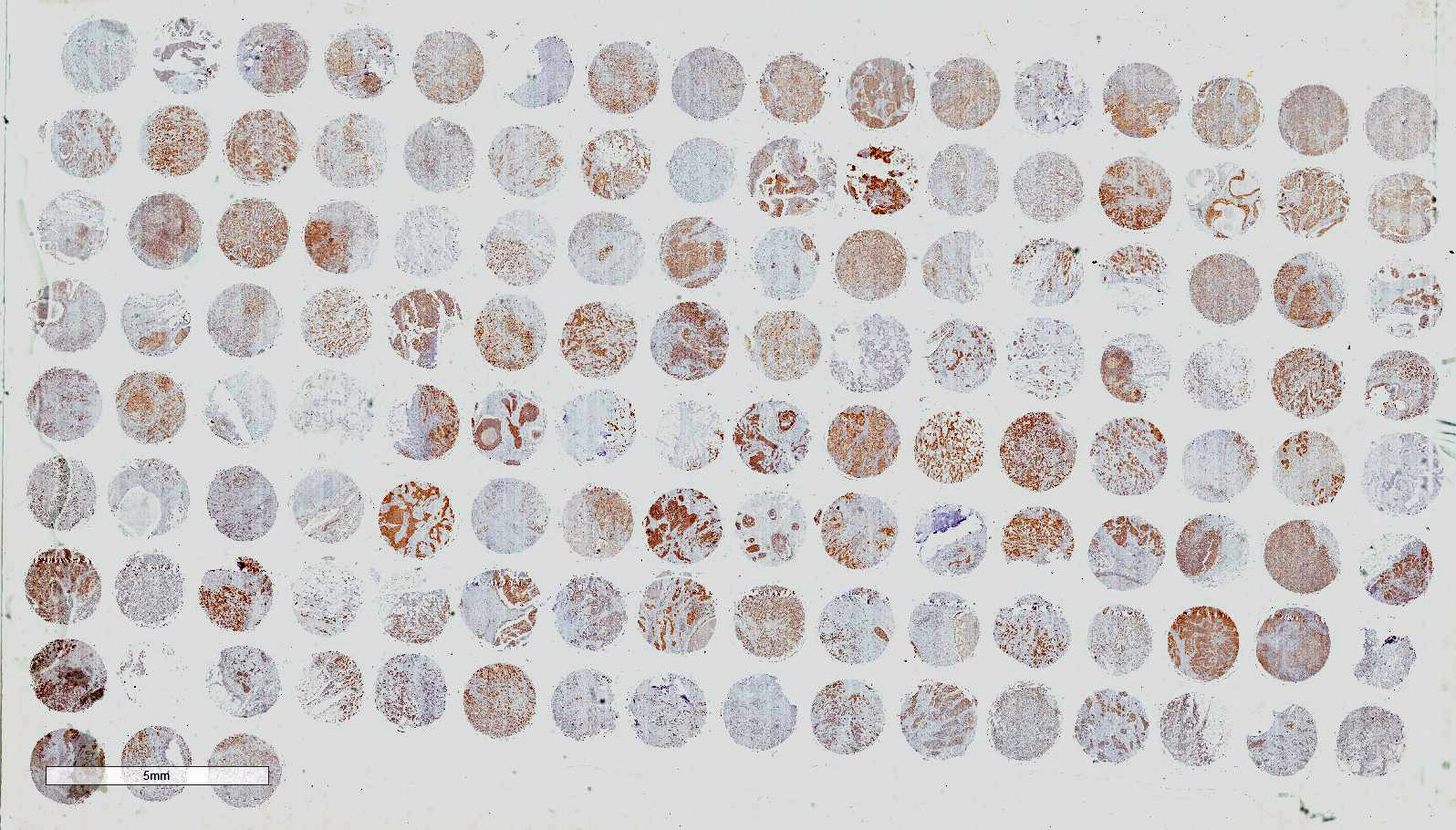

Supplement: Supplementary file 2 [file Image_2.jpeg]
